# miR-203 Expression in Exfoliated Cells of Tongue Coating Represents a Sensitive and Specific Biomarker of Gastroesophageal Reflux Disease

**DOI:** 10.1155/2016/2349453

**Published:** 2016-09-07

**Authors:** Xiuli Yan, Shengliang Zhu, Hui Zhang

**Affiliations:** ^1^Yueyang Hospital of Integrated Traditional Chinese and Western Medicine, Shanghai University of Traditional Chinese Medicine, 110 Ganhe Road, Hongkou, Shanghai 200437, China; ^2^Research Center for Traditional Chinese Medicine Complexity System, Shanghai University of Traditional Chinese Medicine, 1200 Cailun Road, Pudong, Shanghai 201203, China

## Abstract

*Background and Aim*. MicroRNAs (miRNAs) have been implicated in the pathophysiology of numerous human diseases including gastroesophageal reflux disease (GERD). The objective of this study was to investigate the miRNA expression of exfoliated cells of the tongue in patients with GERD versus healthy controls (Ctrls).* Methods*. Using quantitative reverse-transcription PCR (qRT-PCR), expression levels of six candidate miRNAs (miR-143, miR-145, miR-192, miR-194, miR-203, and miR-205) were examined across a discovery cohort of patients with GERD (*n* = 24) versus Ctrls (*n* = 24). These findings were confirmed across a validation cohort (GERD, *n* = 142; Ctrls, *n* = 48). Differences in miRNA expression levels were evaluated using the Mann-Whitney* U* test while the specificity and sensitivity were obtained using receiver-operator characteristic (ROC) curves.* Results*. miR-203 was significantly downregulated in GERD patients as compared to Ctrls (*P* < 0.0001) with ROC curve of 0.94 (95% CI: 0.90–0.97). The sensitivity and the specificity of miR-203 were 91.7% and 87.3%, respectively, in the GERD and Ctrls. These results suggest that miR-203 may be a useful diagnostic marker for discriminating GERD from Ctrls.* Conclusions*. miR-203 testing may assist in the diagnosis of patients with symptoms suggestive of GERD.

## 1. Introduction

Gastroesophageal reflux disease (GERD) is a chronic disease of mucosal damage caused by the backflow of stomach acid into the esophagus. GERD is a condition that knows no boundaries, affecting 5–7% of the population worldwide. It has been shown to have a significant negative impact on the quality of life of those affected, disrupting their daily activities [1]. Diagnostic methods to confirm or reject GERD include empirical PPI treatment, GERD specific questionnaires, endoscopy, and ambulatory reflux monitoring. Unfortunately, these approaches do not achieve high sensitivity and specificity, particularly in patients with nonerosive reflux disease (NERD), and some of them are invasive and expensive [2].

The tongue is increasingly considered, an extension of the upper gastrointestinal tract that can provide important clues to the body's current condition and health status [3]. The fur-like substances covering the surface of the human tongue, called tongue coating, are a very sensitive index of the physiological and pathological status of the organs. The microbiome within the tongue coating has been suggested as a novel holistic biomarker for characterizing gastritis patient subtypes (Cold/Hot Syndromes) [3]. Changes in metabolic patterns and microecological indexes of tongue coating are associated with chronic gastritis [4], while thickening of the tongue coatings and changes in the microbial community structure can be observed in patients with colorectal cancer [5]. Taken together, these studies suggest that tongue coating may represent an important index of body status. Moreover, evaluation of the tongue coating is a relatively noninvasive, simple, safe approach that may yield important contributions to clinical diagnoses.

MicroRNAs (miRNAs) are evolutionarily conserved, small (typically ~22 nucleotides long) RNA molecules that modulate the expression levels of target genes and are thus actively involved in a wide range of physiologic and pathologic processes. As such, numerous studies have highlighted the use of miRNAs as highly sensitive and specific biomarkers for the diagnosis of several diseases [6–9]. In previous studies, a link between gastroesophageal disease and changes in miRNA expression levels was established [10–12]. Specifically, Wijnhoven et al. [10] have shown that miR-203 and miR-205 are high in normal squamous epithelium and low in columnar epithelia, while miR-21, miR-143, miR-145, miR-194, and miR-215 are significantly upregulated in columnar tissues compared with normal squamous epithelium. Smith et al. [11] demonstrated that miR-143, miR-145, and miR-205 expression levels appear to be elevated in oesophageal squamous mucosa of individuals with ulcerative oesophagitis, while Bus et al. [12] reported that miR-143, miR-145, miR-192, and miR-194 were upregulated in esophageal epithelial cells upon acidic bile salt stimulation. Importantly, the miRNA expression in the tongue coating of patients with GERD has not yet been evaluated.

The objective of this study was to investigate the differences of miRNAs expression in exfoliated cells of the tongue coating between patients with GERD and healthy controls (Ctrls). We evaluated the expression levels of miR-143, miR-145, miR-192, miR-194, miR-203, and miR-205 in exfoliated cells of the tongue coating in patients with GERD and Ctrls across a discovery cohort of 48 specimens. We validated our findings across an independent cohort, thereby confirming that miR-203 was significantly downregulated in GERD as compared to Ctrl levels. Our study demonstrated that miR-203 in exfoliated cells of the tongue coating can be used as a potential noninvasive biomarker for the diagnosis of GERD.

## 2. Methods

### 2.1. Study Subjects and Clinical Parameters

From January 2014 to March 2015, 166 patients with GERD were recruited from Shanghai Yueyang Hospital of Integrated Traditional Chinese and Western Medicine. All patients underwent an open-access transoral upper gastrointestinal endoscopy, and those who had typical symptoms (heartburn and regurgitation) or abnormal acid contact time detected at ambulatory esophageal pH testing were considered for inclusion in the study. Exclusion criteria were as follows: (1) GERD combined with other structural gastrointestinal disorders, such as peptic ulcer disease or esophageal or gastric malignancy; (2) prior gastric surgery; and (3) pregnancy. Seventy-two (*n* = 72) healthy normal volunteers who had a physical examination (at Shanghai Yueyang Hospital of Integrated Traditional Chinese and Western Medicine) and health history including question about drug use were recruited and used to represent the normal control group. Only those evaluations (history, physical examinations, and laboratory tests) needed to determine eligibility for a particular study will be done. The clinical parameters of these patients were shown in Table 1. This study was approved by the Institutional Review Board of Shanghai Yueyang Hospital of Integrated Traditional Chinese and Western Medicine. Informed consent was signed by each of the participants, and the study protocol conformed to the ethical guidelines of the Declaration of Helsinki (1964).

### 2.2. Tongue Coating Samples Collection and RNA Isolation

To avoid influence of other possible confounding factors, tongue coating samples were collected in patients with newly diagnosed GERD or first visit. All tongue coatings of GERD patients and healthy volunteers were sampled in the morning prior to breakfast. All participants were required to rinse their mouth before sampling. All tongue coatings were stored at −80°C. RNA was isolated using a mirVana PARIS kit (Ambion, Austin, TX, USA) according to the manufacturer's protocol followed by the treatment of RNase-free DNase I (Promega, Madison, WI, USA) to eliminate DNA contamination.

### 2.3. Quantification of miRNAs

Quantification of miRNAs was based on the stem-loop RT-PCR method. First, a miRNA-specific stem-loop RT primer is hybridized to the miRNA and then reverse transcribed. Next, the RT product is amplified and monitored in real-time using a miRNA-specific forward primer and the universal reverse primer (Table 2). PCR was performed with SYBR Green PCR Master Mixture (TOYOBO, LTD, Japan) according to the manufacturer's instructions by using ABI StepOnePlus real-time PCR system. The specificity of each PCR product was validated by performing melting curve analysis at the end of PCR cycles. All samples were analyzed in triplicate and the cycle threshold (Ct) was defined as the number of cycles required for the fluorescent signal to reach the threshold. The levels of miRNAs were calculated using the formula 2^ΔCt^, where ΔCt = Ct of internal reference −Ct of target miRNA. U6 RNA was used as a miRNA internal control. Data obtained by real-time PCR were translated in log 10 (relative level).

### 2.4. Statistical Analysis

Comparisons between groups were analyzed using Mann-Whitney* U* test. Receiver-operator characteristic (ROC) curves were established to evaluate the difference in the levels of miRNAs among GERD patients and Ctrls. All tests were two-tailed and *P* < 0.05 was considered statistically significant.

## 3. Results 

### 3.1. Description and Clinical Features of the Patient Cohorts

A total of 166 GERD patients and 72 healthy controls were included in this study. Patient demographics are shown in Table 1. All patients were grouped according to the LA GERD classification. 103 participants were classified as grade A (62%), grade B (*n* = 42, 25%), grade C (*n* = 11, 7%), and grade D (*n* = 6, 4%). Only 4 study subjects were diagnosed with Barrett esophagus.

### 3.2. miRNAs Expression in Exfoliated Cells of the Tongue Coating of GERD and Ctrls

**Figure 1:**
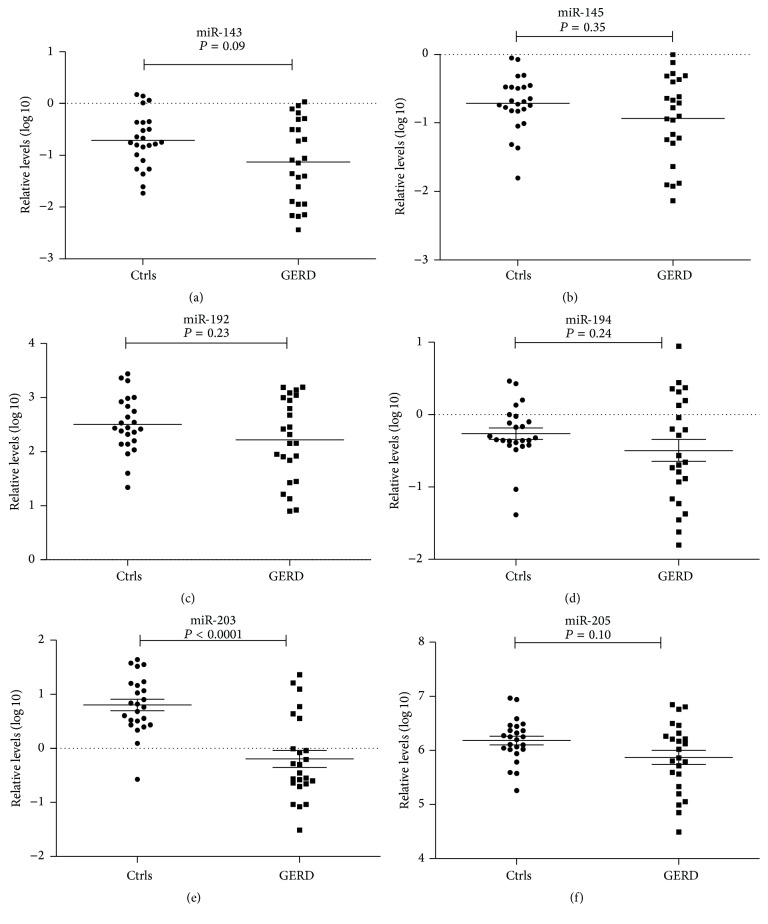
miRNA expression profile in exfoliated cells of the tongue in GERD and Ctrls. qRT-PCR expression levels of miR-143 (a), miR-145 (b), miR-192 (c), miR-194 (d), miR-203 (e), and miR-205 (f) in patients with GERD (*n* = 24) and healthy donors (Ctrls, *n* = 24). The median level of expression is represented in each group by a solid line. The values are normalized to U6 expression levels and displayed in log 10 scale on *Y*-axis.

To assess differential expression of miRNAs in exfoliated cells of the tongue coating between GERD (*n* = 24) and Ctrls (*n* = 24), 6 candidate miRNAs (miR-143, miR-145, miR-192, miR-194, miR-203, and miR-205) were selected. In this manner, we found that miRNAs can be found in exfoliated cells of the tongue coating in Ctrls and patients with GERD. Meanwhile, we observed a statistically significant downregulation of miR-203 in GERD as compared to Ctrls (*P* < 0.0001) (Figure 1).

### 3.3. miR-203 Level in Exfoliated Cells of the Tongue Coating Was Downregulated in Patients with GERD

**Figure 2:**
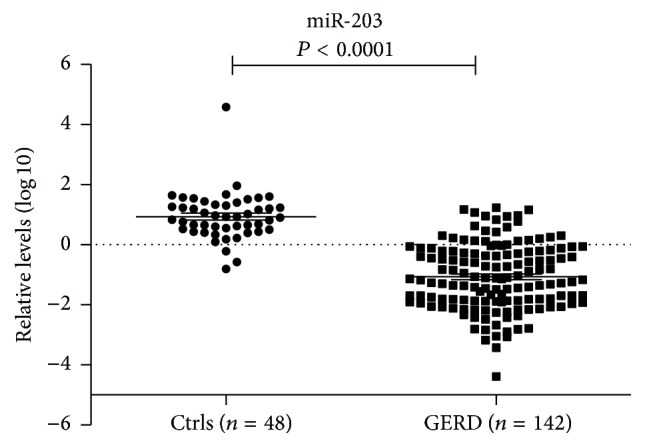
miR-203 expression in patients with GERD and Ctrls. The levels of miR-203 in exfoliated cells of the tongue were evaluated by qRT-PCR in patients with GERD (*n* = 142) and Ctrls (*n* = 48). The median level of expression is represented in each group by a solid line. The values are normalized to U6 expression levels and displayed in log 10 scale on *Y*-axis.

To confirm the specificity and accuracy of our findings we pursued additional qRT-PCR testing across a validation cohort composed of 142 GERD and 48 Ctrl individuals. In keeping with our initial observation, miR-203 was significantly downregulated in GERD specimens as compared to those levels in Ctrls (*P* < 0.0001) (Figure 2).

### 3.4. miR-203 Level in Exfoliated Cells of the Tongue Coating Can Be Used to Distinguish GERD Patients from Ctrls

**Figure 3:**
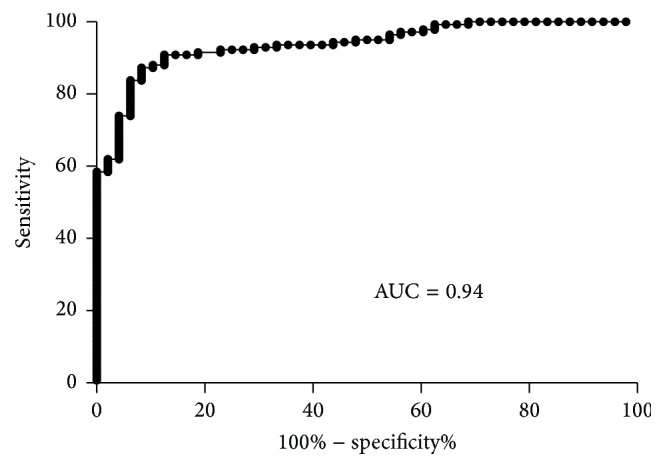
ROC curve analyses. The miR-203 ROC curve was established to discriminate patients with GERD (*n* = 142) from Ctrls (*n* = 48).

Comparing GERD subjects with Ctrls, ROC curve areas of miR-203 were found to be 0.94 (95% CI: 0.90–0.97) (Figure 3). The sensitivity and specificity of miR-203 were 91.7% and 87.3%, respectively, in the GERD subjects and Ctrls. These results suggest that miR-203 can be used to discriminate individuals with GERD from Ctrls.

## 4. Discussion

The tongue is part of digestive system and acts as a very good mirror. The tongue has a coating, which represents the state of digestive function in our body. The tongue coating has been shown to play an important role in reflecting the occurrence, development, and prognosis of numerous diseases [2–4]. The normal tongue coating consists of a thin, white film on the surface of the tongue. In the present study, we demonstrated that the tongue coating in patients with GERD was different from the expected color and pattern observed in Ctrls. Patients with GERD exhibited a yellow tongue coating coloring and frequently displayed eroded tongue coating.

Interestingly, the stability of miRNAs has been readily described not only in tissues or cultured cell lines, but also in human body fluid (plasma, serum, saliva, and urine). Moreover, the expression of miRNA species has been recently shown to correlate with different physiological stages and pathological conditions. As such, some disease-specific profiles can provide crucial diagnostic and prognostic information, highlighting the necessity to exploit miRNAs for possible use as biomarkers [6–9].

Several recent studies have documented the aberrant expression of miRNAs in the pathogenesis of GERD. The expressions of miR-143, miR-145, and miR-205 were significantly higher in oesophageal squamous mucosa from subjects with GERD compared to squamous mucosa from subjects without pathological reflux [11]. Subsequent research found that miR-143, miR-145, miR-192, and miR-194 were also significantly increased in esophageal epithelial cells (Het-1A) upon acidic bile salt stimulation [12]. miR-203 and miR-205 are high in normal squamous epithelium and low in columnar epithelia [10]. Consistent differences in expression levels between Barrett's esophagus (BE) and normal epithelia were noted in multiple studies for miR-203 [13].

To the best of our knowledge, tongue coating miRNAs have not been previously compared between GERD patients and Ctrls. In this study, we hypothesised that miR-143, miR-145, miR-192, miR-194, miR-203, and miR-205 expression might be altered in tongue coating in response to chronic gastroesophageal reflux. Therefore, we evaluated these six miRNAs expression levels from the tongue coating in patients with GERD and Ctrls. qRT-PCR results demonstrated that six miRNAs can be found in exfoliated cells of the tongue coating in patients with GERD and Ctrls. miR-203 in exfoliated cells of the tongue coating was significantly downregulated in GERD patients as compared with Ctrls (*P* < 0.0001) with ROC curve areas of 0.94 (95% CI: 0.90–0.97) and a sensitivity and specificity of 91.7% and 87.3%, respectively, in the GERD subjects and Ctrls. These results suggest that the expression level of miR-203 may be useful a marker for discriminating GERD from Ctrls, with the advantage that this technique is noninvasive and inexpensive.

Studies interrogating the expression of miR-203 have suggested that high-level expression is observed in skin and in the esophagus, an organ sharing anatomical similarities with skin [14]. Similarly, in performing whole mouse embryo in situ hybridization, Yi et al. detected miR-203 signal in the epidermis and the tongue [15]. miR-203 expression has been shown to be dysregulated in several malignancies, including gastric cancer, esophageal cancer, lung cancer, squamous cell carcinoma, and cervical cancer [16–20]. miR-203 is significantly downregulated in Barrett's esophagus (BE) and was also reduced for comparisons of Barrett's high grade dysplasia (HGD), or esophageal adenocarcinoma (EAC) tissues as compared to that in esophageal mucosa in several studies [21, 22]. Our study demonstrated that miR-203 in exfoliated cells of the tongue coating was significantly downregulated in GERD patients as compared with Ctrls. This finding suggests that gastroesophageal reflux alters the expression of miRNAs, including miR-203 identified in our study, in exfoliated cells of the tongue coating. We speculate that, after appropriate validation, this noninvasive test can be used to improve diagnosis of GERD and predict indirectly the efficacy of different treatment methods used for controlling GERD. These miRNAs including miR-203 could target genes and pathways that may contribute to GERD pathogenesis; however, the specific molecular mechanism remains to be determined and awaits further investigation.

Recently, miR-203 has been indicated to play a key role in the regulation of normal immunity or inflammation response [23, 24]. However, we found no difference in miR-203 expression in exfoliated cells of the tongue between GERD patients with inflamed and uninflamed esophageal mucosa.

Our study has some limitations. Most of the GERD patients had a grade of A and B, 62%, 25%, respectively. No difference was found in miR-203 expression between the patients with grades A and B. Another limitation was the failure to recruit patients with non-GERD-related disease. Moreover, as global miRNA expression changes were not assessed it is possible that the expression of other miRNAs may be altered in response to chronic reflux.

In summary, we demonstrated that miR-203 was significantly downregulated in exfoliated cells of the tongue coating in patients with GERD. We suggest that miR-203 testing may assist in the diagnosis of patients with symptoms suggestive of GERD. This may lessen the use of unnecessary antireflux therapy and the need for further invasive and expensive diagnostic methods. Nevertheless, this approach should be further validated in larger cohorts of patients, especially those with non-GERD-related disease, to assess their efficacy and potential applicability in a screening setting.

## Figures and Tables

**Table 1:** Clinical characteristics of GERD and healthy control participants.

Parameters	GERD	Healthy
Individuals (*n*)	166 Grade A (*n* = 103), grade B (*n* = 42), grade C (*n* = 11), grade D (*n* = 6), Barrett's esophagus (BE) (*n* = 4)	72
Male	63	30
Female	103	42
Age (years)	59.28 ± 10.5	58.48 ± 5.98

Ages are given as mean ± S.D.

**Table 2:** Primer sequences for qRT-PCR.

miR-143-FP	ACACTCCAGCTGGGTGAGATGAAGCACTG
miR-143-RP	CTCAACTGGTGTCGTGGAGTCGGCAATTCAGTTGAGGAGCTACA
miR-145-FP	ACACTCCAGCTGGGGTCCAGTTTTCCCAGGA
miR-145-RP	CTCAACTGGTGTCGTGGAGTCGGCAATTCAGTTGAGAGGGATTC
miR-192-FP	ACACTCCAGCTGGGCTGACCTATGAATTG
miR-192-RP	CTCAACTGGTGTCGTGGAGTCGGCAATTCAGTTGAGGGCTGTCA
miR-194-FP	ACACTCCAGCTGGGTGTAACAGCAACTCCA
miR-194-RP	CTCAACTGGTGTCGTGGAGTCGGCAATTCAGTTGAGTCCACATG
miR-203-FP	ACACTCCAGCTGGGGTGAAATGTTTAGGAC
miR-203-RP	CTCAACTGGTGTCGTGGAGTCGGCAATTCAGTTGAGCTAGTGGT
miR-205-FP	ACACTCCAGCTGGGTCCTTCATTCCACCGG
miR-205-RP	CTCAACTGGTGTCGTGGAGTCGGCAATTCAGTTGAGCAGACTCC
URP	TGGTGTCGTGGAGTCG
U6-FP	CTCGCTTCGGCAGCACA
U6-RP	AACGCTTCACGAATTTGCGT
